# Sexual Dysfunction in Chronically Medicated Male Inpatients With Schizophrenia: Prevalence, Risk Factors, Clinical Manifestations, and Response to Sexual Arousal

**DOI:** 10.3389/fpsyt.2021.761598

**Published:** 2022-01-14

**Authors:** Dianying Liu, Shaohua Liu, Meihong Xiu, Hongdong Deng, Huiyun Guo, Wanglin Liu, Delong Zhang, Zhongzhen Mao, Dan Huang, Donghua Huang, Qiumei Miao, Lijuan Qiu, Ning Olivia Zhao, Hanjing Emily Wu, Xiangyang Zhang

**Affiliations:** ^1^Ganzhou City Key Laboratory of Mental Health, The Third People's Hospital of Ganzhou City, Ganzhou, China; ^2^Beijing HuiLongGuan Hospital, Peking University HuiLongGuan Clinical Medical School, Beijing, China; ^3^Department of Psychiatry and Behavioral Sciences, The University of Texas Health Science Center at Houston, Houston, TX, United States; ^4^Institute of Psychology, Chinese Academy of Sciences, Beijing, China; ^5^Department of Psychology, University of Chinese Academy of Sciences, Beijing, China

**Keywords:** schizophrenia, sexual dysfunction, pleasure, arousal, motivation

## Abstract

**Background:**

Sexual dysfunction is a common symptom in patients with schizophrenia, especially in chronically medicated patients. However, the relationship between sexual dysfunction and emotional response to sexual arousal in male patients with schizophrenia remains unclear. This study aimed to assess the incidence, risk factors of sexual dysfunction in males, and their clinical correlations to sexual arousal in male patients with schizophrenia in China.

**Methods:**

A total of 162 male patients, aged 18–50 years, with schizophrenia were recruited from a psychiatric hospital in Ganzhou. The clinical symptoms were assessed by the Positive and Negative Syndrome Scale (PANSS). The Arizona Sexual Experience Scale was utilized to evaluate sexual dysfunction. Erotic images were selected from International Affective Picture System (IAPS). Sixty-eight out of the 162 subjects completed the erotic pictures reactivity task.

**Results:**

Overall, 48 (29.6%) patients were measured as having global sexual dysfunction, 72 (44.4%) patients as having strength of sex drive dysfunction, 51 (31.5%) patients as having sexual arousal dysfunction, 55 (34.0%) patients as having penile erection dysfunction, 60 (37.0%) patients as having reached orgasm dysfunction, and 60 (37.0%) patients as having satisfaction with orgasm dysfunction. The sexual dysfunction patients had significantly higher scores on the negative symptoms of the PANSS. The only important predictor of sexual dysfunction was the severity of PANSS negative factor. The sense of pleasure and arousal post viewing erotic images in the sexual dysfunction group were lower compared to the non-sexual dysfunction group. The sense of pleasure and approach motivation were significantly negatively correlated with the severity of sexual dysfunction.

**Conclusions:**

This study shows that nearly one-third of young and middle-aged chronically medicated male inpatients with schizophrenia suffer from sexual dysfunction. The negative factor of the PANSS can be regarded as the risk factor of sexual dysfunction. Schizophrenia patients with sexual dysfunction experienced lower pleasure and higher avoidance motivation than non-sexual dysfunction patients when exposed to erotic stimuli.

## Introduction

Sexual dysfunction is a serious side effect that commonly manifests in patients with chronic schizophrenia (1–3). Previous studies have shown that the prevalence of global sexual dysfunction in schizophrenia patients ranged from approximately 16% to 96% (4–6). Generally, the illness itself and antipsychotic drugs both can lead to sexual dysfunction in schizophrenia patients (3, 5, 7). Moreover, sexual dysfunction has a negative impact on treatment adherence and intimate relationships, especially in males (3, 6, 8, 9). In addition, due to decreased libido and erectile dysfunction, most males with chronic schizophrenia are accompanied by low self-esteem (10, 11) and depression (3, 12), which reduce their motivation to seek intimacy and damage their marital relationships (13). Therefore, the rate of unmarried patients with schizophrenia is significantly higher when compared to subjects without the disease (14).

A variety of impacting factors, such as sex (14–17), age (12, 14, 18), marital status (14), and smoking status (5, 19, 20), have all been reported to be related to sexual dysfunction in patients with schizophrenia. Moreover, other risk factors for sexual dysfunction include age of onset, duration of illness (15, 17), psychopathology, and prolactin levels (21–24). In addition, antipsychotic medication is a specific and severe risk factor for increased contingency of sexual dysfunction. Some previous studies have supported that long-term use of antipsychotics, such as clozapine, risperidone, and olanzapine, contributed to drug-induced sexual dysfunction (1–3, 5, 7, 14, 17, 25). Although the results regarding sexual dysfunction due to different antipsychotics were inconsistent (7, 17), sexual dysfunction is still regarded as a serious side effect. Some studies have shown that sexual dysfunction is correlated with the severity of clinical symptoms. For example, Huang et al. (6) reported that in a sample of 742 schizophrenia patients, sexual dysfunction was related to the Brief Psychotic Rating Scale (negative domain) score. Similarly, the severity of sexual dysfunction was found to be significantly associated with the negative subscale of the Positive and Negative Syndrome Scale (PANSS) (26). Although most previous studies have examined the association between comorbid sexual dysfunction, demographic variables, and psychopathological symptoms, they did not explore the correlation between sexual dysfunction and emotional response to erotic stimuli (12, 15, 17, 27). Deficits in emotional and motivational function are well-known in patients with schizophrenia, including flat affect, an impaired ability to experience pleasure, and diminished motivation (28). Generally, images depicting erotic contents can evoke a robust motivational state (29). When presenting erotic images, the judgment of pleasure and arousal in individuals with schizophrenia is similar to healthy individuals (30–32). However, the degree of brain activation and desire motivation elicited by erotic stimuli in schizophrenia patients were significantly lower. Heerey and Gold (33) showed that the basic motivational process in patients with schizophrenia may be impaired. However, whether sexual dysfunction is related to the impairment of emotional and motivational systems in schizophrenia patients remains unclear.

The purposes of our study are to explore: (1) the incidence, risk factors, and clinical correlates of sexual dysfunction in Chinese male inpatients with schizophrenia; (2) response to sexual arousal; and (3) the correlation between sexual dysfunction and emotional response to sexual stimuli.

## Methods

### Subjects and Settings

The subjects in the study were inpatients recruited from the Third People's Hospital in Ganzhou City, China. The survey was conducted from August 1, 2018, to July 31, 2019. All subjects met the criteria, as follows: (1) aged 18 years or above, Han Chinese; (2) self-report of heterosexuality; (3) meeting The Diagnostic and Statistical Manual of Mental Disorders IV, Text Revision (DSM-IV-TR) (2000) criteria for schizophrenia, two psychiatrists independently evaluated the mental status of the subjects based on the Structured Clinical Interview for DSM-IV-TR (SCID); (4) adherence to oral second-generation antipsychotics for at least 1 year; (5) be able to understand the requirements and content of the evaluation; (6) no other somatic diseases, such as diseases of the central nervous system, acute, unstable, or life-threatening diseases (e.g., cancer, infection); (7) normal or corrected-to-normal vision and normal color vision. The sociodemographic and clinical information of the subjects was collected by experienced researchers. Other pieces of information were collected from electronic medical records and ancillary resources. All the subjects underwent physical examination and blood biochemical tests. In total, 162 male patients were recruited in this study. All subjects had no addiction to drugs, alcohol, or other substances.

### Ethics Approval and Consent to Participate

The project was approved by the Ethics Committee of the Third People's Hospital of Ganzhou City (2018002). A trained researcher explained the purpose of the study, its procedures, and confidentiality of the information to the subjects in a language appropriate to their level of understanding and emotional state. Participants were also informed that the study was only a cross-sectional survey, that there would be no harm to them, and that they would not obtain any intervention. Each subject signed an informed consent form prior to participation in the study.

All methods described in this study were carried out in accordance with the Declaration of Helsinki promulgated by the National Institutes of Health.

### Clinical Measurement

The PANSS was used by two researchers who received training on how to assess PANSS. The interrater correlation coefficient of repeated measurements for the total score of PANSS was >0.8. The five-factor model of PANSS was used to evaluate the clinical symptoms of the subjects (34). Items P1, P3, P5, and G9 composed positive factor; items N1, N2, N3, N4, N6, and G7 composed negative factor; items G2, G3, and G6 composed depressed factor, which was applied to assess the depressive symptoms; items P2, N5, and G11 composed concrete/disorganized factor; and items P4, P7, G8, and G14 composed excited factor.

### Measurement of Anthropometric Variables

Sociodemographic information included age, age of onset, education levels, marital status at the time of consultation, and family history of mental illness. Clinical data such as weight, height, smoking status, duration of illness, hospitalization numbers, and the use of antipsychotic and antidepressant medications were collected. Trained research assistants were responsible for collecting the sociodemographic and clinical data.

### Sexual Dysfunction

The Mandarin Chinese version of the Arizona Sexual Experience Scale (ASEX) (35–37) was used to assess the sexual functioning of each subject with schizophrenia. The Mandarin Chinese version of ASEX has good reliability and validity in China and has been used in patients with schizophrenia (24, 35, 36, 38, 39). ASEX assessed the status of sexual function in the last week. Five aspects of sexual functioning composed of strength of sexual drive, sexual arousal, penile erection, ability to reach orgasm, and satisfaction with orgasm were measured; each category with a score of Likert 1–6. Total score ranges from 5 to 30, with higher scores indicating much more serious sexual dysfunction. A total score ≥19, any single item score ≥5, or any three item scores ≥4 are defined as the presence of clinical sexual dysfunction.

### Erotic Pictures Reactivity Task

Pictures for the affective reactivity task included 10 erotic images of nude couples in erotic poses and 10 neutral images of dressed people in daily life. The pictures were all selected from the International Affective Picture System (IAPS) and have been used in previous studies (40–42). According to the normative rating program provided by Bradley et al. (29) in the IAPS manual, E-prime was used to edit the evaluation program. After providing instruction consent, the 20 pictures were presented to participants in a random order. The participants watched each image for 6 s, and the subjects were asked to rate pleasure, arousal, and approach motivation at the same time through a computer. Participants rated three dimensions with self-report in a 9-point rating scale. The pleasure dimension is rated as 1 indicating “extreme disgust,” 5 indicating “neutral,” and 9 indicating “extreme pleasure.” Similarly, in the arousal dimension, 1 indicates “not at all arousing,” 5 indicates “moderately arousing,” and 9 indicates “extremely arousing.” Approach motivation evaluated the degree to which subjects want to get involved in the scene or approaching the scene after viewing pictures, with 1 indicating “extremely escaping,” 5 indicating “neutral,” and 9 indicating “extremely approaching.” Before the formal experiment, participants were given practice lists in order to understand the scoring standard. During the formal experiment, the subject was left alone in a private room.

### Data Analysis

The G^*^Power 3.1.9.2 program was used to perform a sample size based on effect size derived from a previous study (26). The sociodemographic and clinical variables of sexual dysfunction (SD) and non-sexual dysfunction (NSD) groups were compared. The continuous variables were tested by independent-samples *t*-test, and the categorical variables were compared by chi-square test. The prevalence of global sexual dysfunction and each factor were analyzed by chi-square test. ANCOVA was performed to control the influence of age, education years, age of onset, hospitalization numbers, and family history of mental illness. Correlations of sexual dysfunction and sociodemographic and clinical variables were conducted by Pearson correlation coefficients. Bonferroni corrections were used to adjust for multiple tests. Then, a stepwise multiple regression analysis was applied to explore significantly predictive variables correlated to sexual dysfunction.

The pleasure, arousal, and approach motivation ratings were analyzed with 2 × 2 mixed-model analyses of variance (ANOVAs), and the Greenhouse–Geisser method was run to adjust degrees of freedom. Using the type of pictures (erotic picture and neutral picture) as within-subject factors and sexual dysfunction (NSD and SD)as between-subject factors.

SPSS 22.0 was conducted for all statistical analyses. *p*-values were double-tailed with significance level at 0.05. The continuous variables were presented in the form of mean ± standard deviation.

## Results

### Demographic and Clinical Characteristics of Patients

Based on the effect sizes derived from a previous study (26), the sample size should be 96. Thus, 162 patients in the present study were sufficient to obtain a power of 0.8 at α <0.05 (two-tailed). Of the 162 male chronic schizophrenia patients, 128 were single (79.0%) and 34 were married (21.0%). The age of the patients ranged from 18 to 50 years, with an average age of 28.41 years (standard deviation = 7.61). The duration of education ranged from 5 to 16 years, with an average of 9.29 years (standard deviation = 2.74). The age of onset ranged from 7 to 48 years old, with an average of 22.12 years (standard deviation = 7.20). The duration of illness ranged from 1 to 35 years, with an average of 6.51 years (standard deviation = 5.92). Hospitalization ranged from 1 to 11 times, with an average of 3.88 times (standard deviation = 2.70). The average body mass index (BMI) was 22.70 (standard deviation = 3.80), ranging from 14.69 to 33.31. Seventy-nine patients (48.8%) were active smokers, and 32 patients (19.8%) had a family history of psychiatric disorders.

### Prevalence of Sexual Dysfunction in Male Patients With Chronic Schizophrenia

The prevalence of global sexual dysfunction was 29.6% (48/162) in male patients with chronic schizophrenia, strength of sex drive dysfunction was 44.4% (72/162), sexual arousal dysfunction was 31.5% (51/126), penile erection dysfunction was 34.0% (55/162), the ability to reach orgasm dysfunction and satisfaction with orgasm dysfunction was 37.0% (60/162). In addition, there were significant group differences in each domain of ASEX, including strength of sex drive, sexual arousal, penile erection, ability to reach orgasm, satisfaction with orgasm, and total score (all *p* < 0.05) (Table 1).

**Table 1:** Frequency of sexual dysfunction in each domain of ASEX.

**ASEX Items**	***N* (%)**	**SD patients**	**NSD patients**	***t***	***p***
Strength of sex drive	72/162(44.4)	4.94 ± 0.87	2.60 ± 0.0.63	19.84	<0.001
Sexual arousal	51/162(31.5)	4.86 ± 0.90	2.40 ± 0.74	18.39	<0.001
Penile erection	55/162(34.0)	4.87 ± 0.88	2.49 ± 0.64	19.76	<0.001
Ability to reach orgasm	60/162(37.0)	5.08 ± 0.91	2.43 ± 0.68	21.09	<0.001
Satisfaction with orgasm	60/162(37.0)	5.03 ± 0.90	2.31 ± 0.68	21.82	<0.001
Total score ≥19	48/162(29.6)	24.4 ± 4.05	13.67 ± 2.82	19.30	<0.001

*Scores of 4 or greater on the 1–6 Likert scale were considered “dysfunctional”*.

*Individual with one or more of the following: total ASEX score ≥19, score on any of 5 items ≥5; score on 3 or more items ≥4*.

*SD, sexual dysfunction group; NSD, non- sexual dysfunction group*.

### Comparison of Demographic and Clinical Variables Between Sexual Dysfunction and Non-sexual Dysfunction Patients

Sociodemographic and clinical variables of all patients are shown in Table 2. Compared with non-sexual dysfunction patients, sexual dysfunction patients had significantly higher negative symptom of PANSS (*p* < 0.01). After controlling for age and age of onset, a significant difference was observed in negative symptoms (*F* = 5.30, *p* < 0.05) between non-sexual dysfunction and sexual dysfunction groups.

**Table 2:** Characteristics of schizophrenia patients with or without sexual dysfunction ASEX.

	**All patients (*N =* 162)**	**SD patients (*N =* 48)**	**NSD patients (*N =* 114)**	***t* or *x*^2^**	***p***
Age (years)	28.41 ± 7.61	27.98 ± 8.73	28.56 ± 7.18	0.44	0.66
Education (years)	9.29 ± 2.74	8.98 ± 3.60	9.07 ± 2.65	0.17	0.87
**Marital status**					
Single/married	128/34 79.01%/20.99%	41/7 85.42%/14.58%	87/27 76.32%/23.68%	1.68	0.19
Family history of mental illness (yes/no)	32/130 19.75%/80.25%	11/37 22.92%/77.08%	21/93 18.42%/81.58%	0.51	0.52
Smoking behavior (yes/no)	79/83 48.77%/51.23%	18/30 37.50%/62.50%	61/53 53.51%/46.49%	0.06	0.09
BMI (kg/m^2^)	22.70 ± 3.80	22.49 ± 4.24	22.78 ± 3.63	0.45	0.66
Duration of illness (years)	6.51 ± 5.92	6.08 ± 6.09	6.54 ± 6.01	0.44	0.66
Age of onset (years)	22.12 ± 7.20	22.23 ± 8.22	22.20 ± 6.99	−0.02	0.99
Hospitalization numbers	3.88 ± 2.70	2.20 ± 3.38	3.70 ± 4.45	1.95	<0.05
**Psychopathology PANSS**					
Positive symptom	18.31 ± 6.64	18.27 ± 5.67	18.33 ± 7.03	0.55	0.96
Negative symptom	18.72 ± 7.80	21.02 ± 8.81	17.75 ± 7.15	−2.48	<0.01
General psychopathology	39.48 ± 11.71	40.90 ± 10.65	38.89 ± 12.13	−1.00	0.32
Total score	76.51 ± 22.36	80.19 ± 21.47	74.96 ± 22.64	−1.36	0.18
Positive factor	11.69 ± 7.28	12.31 ±5.40	11.43 ± 5.23	−0.97	0.33
Negative factor	15.28 ± 7.36	18.00 ± 8.24	14.14 ± 6.66	−3.13	<0.01
Cognitive factor	8.38 ± 3.38	8.17 ± 3.61	8.46 ± 3.30	0.51	0.61
Excited factor	9.13 ± 3.90	8.60 ± 3.60	9.35 ± 4.02	1.11	0.27
Depressive factor	7.31 ± 3.20	7.54 ± 3.27	7.21 ± 3.18	−0.60	0.55
**On antipsychotics**				0.54	0.80
Clozapine, (*n*, %)	46(28.40)	13(27.08)	33(28.95)		
Risperidone, (*n*, %)	97(59.87)	28(58.34)	69(60.52)		
SGAs except Clozapine and Risperidone, (*n*, %)	19(11.73)	7(14.58)	12(10.53)		
On antidepressants, (*n*, %)	8(4.94)	2(4.17)	6(5.26)	0.09	0.77

### The Effect of Antipsychotics on Sexual Dysfunction

Among all participants, the most frequently used antipsychotic drug was risperidone [97 (59.87%)], followed by clozapine [46 (28.40%)] and other second-generation antipsychotics [19 (11.73%), e.g., olanzapine 14, quetiapine 5]. ANOVA showed no significant effect of antipsychotics on sexual dysfunction (all *p* > 0.05) (Table 3). Eight participants were prescribed citalopram in their medications. There was no significant effect of antidepressant on sexual dysfunction (all *p* > 0.05) (Table 2).

**Table 3:** The effect of antipsychotics on sexual dysfunction ASEX.

**ASEX Items**	**Clozapine**	**Risperidone**	**Olanzapine**	***F***	***P***
Strength of sex drive	3.74 ± 1.54	3.60 ± 1.38	3.63 ± 1.01	0.929	0.379
Sexual arousal	3.07 ± 1.50	3.24 ± 1.40	3.11 ± 1.15	0.051	0.950
Penile erection	3.48 ± 1.47	3.25 ± 1.32	3.11 ± 1.20	1.051	0.352
Ability to reach orgasm	3.28 ± 1.54	3.43 ± 1.46	3.63 ± 1.61	0.628	0.535
Satisfaction with orgasm	3.17 ± 1.66	3.34 ± 1.44	3.58 ± 1.61	1.091	0.338
Total score	16.74 ± 6.77	16.86 ± 5.69	17.05 ± 4.63	0.458	0.634

### Correlation of Sexual Dysfunction and Demographic and Clinical Measures

Pearson correlation analysis showed that sexual dysfunction was correlated with negative symptoms (*r* = 0.16, *p* = 0.04; Bonferroni corrected *p* > 0.05). Furthermore, a multiple stepwise regression was performed to predict sexual dysfunction with all sociodemographic and clinical variables. Only negative symptoms predicted sexual dysfunction in a statistically significant manner [*F*_(1,160)_ = 6.39, *p* < 0.01, adjusted *R*^2^ = 0.03]. The coefficients of the variable are shown in Table 4.

**Table 4:** Predictors generated by Multivariate Logistic Regression with ASEX total score as dependent variables.

	**Coefficients**		**Standardized coefficients**	***t***	***p***	**95.0% confidence interval for** ***B***	
	***B***	**S. E**	**Beta**			**Lower bound**	**Upper bound**
(Constant)	14.452	1.050		13.766	<0.001	12.379	16.526
Negative factor	0.157	0.062	0.198	2.529	0.012	0.034	0.279

### Response to Erotic and Neutral Pictures Between Sexual Dysfunction and Non-sexual Dysfunction Patients

Sixty-eight subjects were administered the affective reactivity tasks. The pleasure, arousal, and approach motivation of the erotic pictures and neutral pictures in each group are shown in Table 5. Two-way ANOVAs showed significant interaction effects between sexual dysfunction group (SD vs. NSD) and picture types (Erotic Picture vs. Neutral Picture) on pleasure and arousal evaluation (all *p* < 0.05). However, there was no significant effect of sexual dysfunction group, picture types, and sexual dysfunction group × picture types on approach motivation evaluation (Table 5).

**Table 5:** Response to erotic and neutral picture between sexual dysfunction and non-sexual dysfunction patients.

	**SD Group**		**NSD group**		**Group**		**Picture type**		**Group** **×** **Picture**	
	**Erotic picture**	**Neutral picture**	**Erotic picture**	**Neutral picture**	***F***	***P***	***F***	***P***	***F***	***P***
Pleasure	4.56 ± 2.07	5.49 ± 1.47	5.56 ± 2.15	5.34 ± 1.89	0.92	0.34	1.89	0.176	4.97	0.029
Arousal	4.73 ± 2.15	5.34 ± 1.40	5.27 ± 2.19	4.80 ± 1.77	0.01	0.989	0.08	0.775	5.15	0.027
Approach Motivation	4.38 ± 2.35	5.09 ± 1.52	5.22 ± 2.21	5.07 ± 1.92	0.78	0.379	1.08	0.303	2.61	0.11

*SD, sexual dysfunction group; NSD, non-sexual dysfunction group*.

### Correlation of Sexual Dysfunction and Response to Erotic Picture

Pearson correlation analysis showed that there was a significantly negative correlation between the severity of sexual dysfunction and the scores of pleasure (*r* = −0.28, *p* = 0.02) and approach motivation (*r* = −0.28, *p* = 0.02) on sexual picture, and all passed the Bonferroni correction.

## Discussion

To the best of our knowledge, this is the first study to investigate the prevalence of sexual dysfunction and its related risk factors among young and middle-aged male Chinese medicated chronic inpatients with schizophrenia, as well as the relationship between sexual dysfunction and the response to erotic pictures. The main findings of this study included: (1) the percentage of global sexual dysfunction was 29.6% in young and middle-aged male medicated chronic inpatients with schizophrenia; (2) negative symptoms were a risk factor for sexual dysfunction; (3) sexual dysfunction patients experienced lower pleasure and approach motivation when viewing erotic pictures.

Our cross-sectional clinical study indicated that nearly one-third of young and middle-aged chronically medicated male inpatients with schizophrenia suffer from sexual dysfunction, which was lower than those of the previous studies that used ASEX in schizophrenia patients in different sociocultural environments (30.7–84.5%) (2, 15, 26, 38, 43) but significantly higher compared to that of the general population, which was estimated between 8 and 15% (8, 15). In a previous study in China, the incidence of sexual dysfunction was 60.7% in male schizophrenia patients treated in a primary care setting (38), while it was 46% in outpatients with schizophrenia in Turkey males (15). Because sex is a private issue, sexual culture and sexual attitude are more conservative in Asia than in western countries. Talking about sex can make subjects feel shame, and reporting sexual dysfunction can damage their self-esteem. Therefore, people in Asia, especially in rural areas, are more likely to underestimate their sexual dysfunction (6, 44). In addition, age is another important risk factor for sexual dysfunction in the general population (45) and schizophrenia patients (15, 38). In our study, the mean age was 28.4 years, which was younger than those in the study by Hou et al. (38) and other studies. Another important factor was the type of antipsychotic. In our study, the second-generation antipsychotic drugs were used by all patients, and there were no significant differences in the effects of antipsychotic and antidepressant drugs on sexual dysfunction, which is consistent with a previous study showing that no differences were found in the prevalence of sexual dysfunction between the drug groups (olanzapine, risperidone, aripiprazole, haloperidol) (6, 43). However, in other studies, there were significant differences in the incidence of sexual dysfunction between different antipsychotic drugs (7, 46, 47). Taken together, the diverse rates of sexual dysfunction are likely to be related to the patients' treatment setting (inpatient, community or outpatient), sample size, different sociocultural contexts, clinical symptoms (especially negative symptoms), and possibly antipsychotic treatment.

Most previous studies have shown that sexual dysfunction is correlated with the severity of psychopathology, and higher scores on the negative symptoms were correlated with the severity of sexual dysfunction (6, 48). In our present study, multiple regression analysis showed that sexual dysfunction was positively correlated with negative symptoms of PANSS, which is consistent with the results of a previous study (26). Malik et al. (49) reported that negative symptoms predicted decreased libido. In addition, our present study did not observe the relationship between sexual dysfunction and duration of illness and age of onset, which was consistent with a previous study (6).

Notably, in our present study, compared with the non-sexual dysfunction group, the sexual dysfunction group showed lower pleasure and arousal induced by sexual stimuli. Furthermore, our study also found that sexual dysfunction was negatively correlated with pleasure and approach motivation elicited by erotic pictures, which is in line with previous investigations that indicated that hedonic response and intrinsic motivation were impaired in schizophrenia (33, 50–52). As pointed out by Najas-Garcia et al. (53), anhedonia, apathy, avolition, and motivational deficits are negative symptoms in schizophrenia, and sexual dysfunction was significantly correlated with negative symptoms. Compared with non-sexual dysfunction patients, when presenting erotic stimuli, schizophrenia patients with sexual dysfunction experienced lower pleasure and sexual arousal and enhanced motivation to avoid sexual stimulation (53). In addition, sexual dysfunction has a negative effect on the self-esteem of schizophrenia patients, reduces the motivation of actively seeking and maintaining intimate relationships, and worsens their quality of life (44, 54).

Our study had several limitations. First, as a cross-sectional study, no causal relationship of sexual dysfunction can be concluded. Second, the sexual dysfunction and emotion were all self-assessment, which may lead to biased results. Third, this study may not be representative because most of the participants in the current study were young and middle-aged men, and the average age of subjects was younger (28.41 years). In addition, our results were only applicable to hospitalized patients, and our findings may not be generalized to other subjects recruited from other locations in China. “Fourth, although we did not find significant differences in the effects of antipsychotic drugs on sexual dysfunction, we did not collect the antipsychotic dose data. Also, we did not measure medication adherence. Therefore, we do not know whether there was a correlation between antipsychotic dose or medication adherence and sex dysfunction in this study, which should be remedied in future investigation.” Fifth, the subjectivity of the responses should be considered as a limitation. In this study, the mean age of our patients was below 30, and for them, reporting sexual dysfunction might be an embarrassment. Moreover, some patients might have thought that the study would lead to a treatment for sexual dysfunction, since we did not write this down on the consent form. Sixth, homosexual preferences would affect response to the erotic stimuli and also the reporting of the response. In this study, whether a patient was heterosexual or non-heterosexual only depended on his self-report, and we did not have any objective measure. In particular, Chinese men are reluctant to admit non-heterosexuality, even if they are homosexual. Therefore, the real impact of homosexual preference to the erotic stimuli and reported responses warrant further investigation. Seventh, adherence to oral second-generation antipsychotics for at least 1 year, it is unclear that patients on treatment for 1 year is considered as chronic as one on treatment for 20 years, where inevitably long-term effects are added to sexual dysfunction. Finally, the sample size was small, especially the numbers of affective reactivity task analysis, which may limit the reliability of the findings.

In conclusion, this study revealed that the prevalence of global sexual dysfunction was higher in young and middle-aged male schizophrenia patients than that in the general population. The negative symptom PANSS was the risk factor of sexual dysfunction. Furthermore, upon exposure to sexual stimulation, male schizophrenia patients with sexual dysfunction experienced lower pleasure and approach motivation. Thus, our findings in this study may provide new insights into understanding the psychopathological mechanism of sexual dysfunction so as to find potential solutions for sexual dysfunction in patients with schizophrenia. Clinicians should assess the sexual functioning of patients with schizophrenia and recommend appropriate antipsychotic medications in combination with physical therapy to improve negative symptoms, such as repetitive transcranial magnetic stimulation (rTMS) and transcranial direct current stimulation (tDCS). In addition, clinicians should implement concurrent psychotherapy to improve medication adherence, self-esteem, and self-confidence; promote interpersonal communications; adjust attitudes; and improve sexual experiences.

## Data Availability Statement

The original contributions presented in the study are included in the article/supplementary material, further inquiries can be directed to the corresponding author/s.

## Ethics Statement

The studies involving human participants were reviewed and approved by the Ethics Committee of the Third People's Hospital of Ganzhou City. The patients/participants provided their written informed consent to participate in this study.

## Author Contributions

XZ: conceptualization, methodology, writing—reviewing, editing, and supervision. DL: formal analysis, writing—original draft, and funding acquisition. HD, HG, WL, DZ, ZM, DaH, DoH, QM, and LQ: investigation, resources, and data curation. SL, MX, NZ, and HW: conceptualization, writing—review, and editing. All authors have contributed to and have approved the submitted version.

## Funding

This research was supported by Key R&D Program of Jiangxi Province (20202BBGL73106), Key R&D Program of Science and Technology in Ganzhou (201926), the Planning Program of Health Committee of Ganzhou (201911), the Planning Program of Health Committee of Jiangxi Province (20202089), and grants 81771139 and 81371477 from the CAS Pioneer Hundred Talents Program and the National Natural Science Foundation of China.

## Conflict of Interest

The authors declare that the research was conducted in the absence of any commercial or financial relationships that could be construed as a potential conflict of interest.

## Publisher's Note

All claims expressed in this article are solely those of the authors and do not necessarily represent those of their affiliated organizations, or those of the publisher, the editors and the reviewers. Any product that may be evaluated in this article, or claim that may be made by its manufacturer, is not guaranteed or endorsed by the publisher.

## References

[1] Montejo AL Montejo L Navarro-Cremades F. Sexual side-effects of antidepressant and antipsychotic drugs. Curr Opin Psychiatry. (2015) 28:418–23. 10.1097/YCO.0000000000000198 26382168

[2] Fanta T Haile K Abebaw D Assefa D Hibdye G. Assessment of sexual dysfunction and associated factors among patients with schizophrenia in Ethiopia, 2017. BMC Psychiatry. (2018) 18:158. 10.1186/s12888-018-1738-3 29843656 PMC5975573

[3] Montejo AL Montejo L Baldwin DS. The impact of severe mental disorders and psychotropic medications on sexual health and its implications for clinical management. World Psychiatry. (2018) 17:3–11. 10.1002/wps.20509 29352532 PMC5775119

[4] Parish WL Luo Y Stolzenberg R Laumann EO Farrer G Pan S. Sexual practices and sexual satisfaction: a population based study of Chinese urban adults. Arch Sex Behav. (2007) 36:5–20. 10.1007/s10508-006-9082-y 17187219

[5] Uçok A Incesu C Aker T Erkoç S. Sexual dysfunction in patients with schizophrenia on antipsychotic medication. Eur Psychiatry. (2007) 22:328–33. 10.1016/j.eurpsy.2007.01.001 17344032

[6] Huang YH Hou CL Ng CH Chen X Wang QW Huang ZH. Sexual dysfunction in Chinese rural patients with schizophrenia. BMC Psychiatry. (2019) 19:218. 10.1186/s12888-019-2205-5 31299942 PMC6624902

[7] Just MJ. The influence of atypical antipsychotic drugs on sexual function. Neuropsychiatr Dis Treat. (2015) 11:1655–61. 10.2147/NDT.S84528 26185449 PMC4501246

[8] Acuña MJ Martín JC Graciani M Cruces A Gotor F. A comparative study of the sexual function of institutionalized patients with schizophrenia. J Sex Med. (2010) 7:3414–23. 10.1111/j.1743-6109.2010.01832.x 20456629

[9] Basson R Gilks T. Women's sexual dysfunction associated with psychiatric disorders and their treatment. Womens Health (Lond). (2018) 14:1745506518762664. 10.1177/1745506518762664 29649948 PMC5900810

[10] Chan KK Mak WW. The mediating role of self-stigma and unmet needs on the recovery of people with schizophrenia living in the community. Qual Life Res. (2014) 23:2559–68. 10.1007/s11136-014-0695-7 24756436

[11] Huguelet P Mohr S Miserez C Castellano P Lutz C Boucherie M. An exploration of sexual desire and sexual activities of women with psychosis. Community Ment Health J. (2015) 51:229–38. 10.1007/s10597-014-9768-x 25064089

[12] Olisah VO Sheikh TL Abah ER Mahmud-Ajeigbe AF. Sociodemographic and clinical correlates of sexual dysfunction among psychiatric outpatients receiving common psychotropic medications in a Neuropsychiatric Hospital in Northern Nigeria. Niger J Clin Pract. (2016) 19:799–806. 10.4103/1119-3077.180063 27811454

[13] Grinshpoon A Ponizovsky AM. The relationships between need profiles, clinical symptoms, functioning and the well-being of inpatients with severe mental disorders. J Eval Clin Pract. (2008) 14:218–25. 10.1111/j.1365-2753.2007.00836.x 18093104

[14] Lee JY Kim SW Lee YH Kang HJ Kim SY Bae KY. Factors associated with self-rated sexual function in Korean patients with schizophrenia receiving risperidone monotherapy. Hum Psychopharmacol. (2015) 30:416–24. 10.1002/hup.2489 26123060

[15] Hocaoglu C Celik FH Kandemir G Guveli H Bahceci B. Sexual dysfunction in outpatients with schizophrenia in Turkey:a cross-sectional study. Shanghai Arch Psychiatry. (2014) 26:347–56. 10.11919/j.issn.1002-0829.214101 25642109 PMC4311108

[16] da Silva TL Ravindran AV. Contribution of sex hormones to gender differences in schizophrenia: a review. Asian J Psychiatr. (2015) 18:2–14. 10.1016/j.ajp.2015.07.016 26321672

[17] Zhao S Wang X Qiang X Wang H He J Shen M. Is there an association between schizophrenia and sexual dysfunction in both sexes? A systematic review and meta-analysis. J Sex Med. (2020) 17:1476–88. 10.1016/j.jsxm.2020.03.005 32299716

[18] Brotto LA Basson R Luria M. A mindfulness-based group psychoeducational intervention targeting sexual arousal disorder in women. J Sex Med. (2008) 5:1646–59. 10.1111/j.1743-6109.2008.00850.x 18507718

[19] Gades NM Nehra A Jacobson DJ McGree ME Girman CJ Rhodes T. Association between smoking and erectile dysfunction: a population-based study. Am J Epidemiol. (2005) 161:346–51. 10.1093/aje/kwi052 15692078

[20] Salokangas RK Honkonen T Stengård E Koivisto AM Hietala J. Cigarette smoking in long-term schizophrenia. Eur Psychiatry. (2006) 21:219–23. 10.1016/j.eurpsy.2005.07.008 16360309

[21] Smith SM O'Keane V Murray R. Sexual dysfunction in patients taking conventional antipsychotic medication. Br J Psychiatry. (2002) 181:49–55. 10.1192/bjp.181.1.49 12091263

[22] Nakonezny PA Byerly MJ Rush AJ. The relationship between serum prolactin level and sexual functioning among male outpatients with schizophrenia or schizoaffective disorder: a randomized double-blind trial of risperidone vs. quetiapine. J Sex Marital Ther. (2007) 33:203–16. 10.1080/00926230701267829 17454518

[23] Rubio-Abadal E Del Cacho N Saenz-Navarrete G Arranz B Cambra RM Cuadras D. How hyperprolactinemia affects sexual function in patients under antipsychotic treatment. J Clin Psychopharmacol. (2016) 36:422–8. 10.1097/JCP.0000000000000539 27433851

[24] Zhang Y Tang Z Ruan Y Huang C Wu J Lu Z. Prolactin and thyroid stimulating hormone (TSH) levels and sexual dysfunction in patients with schizophrenia treated with conventional antipsychotic medication: a cross-sectional study. Med Sci Monit. (2018) 24:9136–43. 10.12659/MSM.913759 30554232 PMC6319142

[25] Souaiby L Kazour F Zoghbi M Bou Khalil R Richa S. Sexual dysfunction in patients with schizophrenia and schizoaffective disorder and its association with adherence to antipsychotic medication. J Ment Health. (2020) 29:623–30. 10.1080/09638237.2019.1581333 30862199

[26] Esan O Esan A. Sexual dysfunction among patients with schizophrenia in Southwest Nigeria. J Sex Marital Ther. (2018) 44:657–66. 10.1080/0092623X.2018.1447055 29509076

[27] Bushong ME Nakonezny PA Byerly MJ. Subjective quality of life and sexual dysfunction in outpatients with schizophrenia or schizoaffective disorder. J Sex Marital Ther. (2013) 39:336–46. 10.1080/0092623X.2011.606884 23421823

[28] Yee CM Mathis KI Sun JC Sholty GL Lang PJ Bachman P. Integrity of emotional and motivational states during the prodromal, first-episode, and chronic phases of schizophrenia. J Abnorm Psychol. (2010) 119:71–82. 10.1037/a0018475 20141244 PMC2853376

[29] Bradley MM Codispoti M Cuthbert BN Lang PJ. Emotion and motivation I: defensive and appetitive reactions in picture processing. Emotion. (2001) 1:276–98. 10.1037/1528-3542.1.3.276 12934687

[30] Hempel RJ Tulen JH van Beveren NJ van Steenis HG Mulder PG Hengeveld MW. Physiological responsivity to emotional pictures in schizophrenia. J Psychiatr Res. (2005) 39:509–18. 10.1016/j.jpsychires.2004.11.004 15992560

[31] Taylor SF Phan KL Britton JC Liberzon I. Neural response to emotional salience in schizophrenia. Neuropsychopharmacology. (2005) 30:984–95. 10.1038/sj.npp.1300679 15689961

[32] Rockstroh B Junghöfer M Elbert T Buodo G Miller GA. Electromagnetic brain activity evoked by affective stimuli in schizophrenia. Psychophysiology. (2006) 43:431–9. 10.1111/j.1469-8986.2006.00424.x 16965604

[33] Heerey EA Gold JM. Patients with schizophrenia demonstrate dissociation between affective experience and motivated behavior. J Abnorm Psychol. (2007) 116:268–78. 10.1037/0021-843X.116.2.268 17516760

[34] Wallwork RS Fortgang R Hashimoto R Weinberger DR Dickinson D. Searching for a consensus five-factor model of the Positive and Negative Syndrome Scale for schizophrenia. Schizophr Res. (2012) 137:246–50. 10.1016/j.schres.2012.01.031 22356801 PMC3351536

[35] McGahuey CA Gelenberg AJ Laukes CA Moreno FA Delgado PL McKnight KM. The Arizona Sexual Experience Scale (ASEX): reliability and validity. J Sex Marital Ther. (2000) 26:25–40. 10.1080/009262300278623 10693114

[36] Byerly MJ Nakonezny PA Fisher R Magouirk B Rush AJ. An empirical evaluation of the Arizona sexual experience scale and a simple one-item screening test for assessing antipsychotic-related sexual dysfunction in outpatients with schizophrenia and schizoaffective disorder. Schizophr Res. (2006) 81:311–6. 10.1016/j.schres.2005.08.013 16298106

[37] de Boer MK Castelein S Wiersma D Schoevers RA Knegtering H. A systematic review of instruments to measure sexual functioning in patients using antipsychotics. J Sex Res. (2014) 51:383–9. 10.1080/00224499.2013.865111 24754359

[38] Hou CL Zang Y Rosen RC Cai MY Li Y Jia FJ. Sexual dysfunction and its impact on quality of life in Chinese patients with schizophrenia treated in primary care. Compr Psychiatry. (2016) 65:116–21. 10.1016/j.comppsych.2015.11.002 26773999

[39] Qin F Ye X Wei H Wen Y Shi L Zhen L. Sexual experience and stigma among chinese patients with an enterostomy: a cross-sectional, descriptive study. Wound Manag Prev. (2019) 65:22–30. 10.25270/wmp.2019.12.2230 31887105

[40] Liu DY Liu MF Zhao LN. Sex differences in response to sexual pictures in heterosexual university students. Chin J Clin Psychol. (2010) 18:607–9+13 10.3877/j.issn.1672-1993.2010.07.01233967908

[41] Liu DY LMR Xie XD Zhong H Zhang B Cai LX Deng G. A study on differences of cognitive mechanism in processing different types of sexual picture in female university students. Chin J Behav Med Brain Sci. (2012) 453–5. 10.3760/cma.j.issn.1674-6554.2012.05.021

[42] Liu DY Liu MF Liu JP. A study on the differences in ERP during sexual arousal elicited by different types of sexual picture in adult men. Chin J Behav Med Brain Sci. (2013) 22:923–5. 10.3760/cma.j.issn.1674-6554.2013.10.019

[43] Johnsen E Kroken R Løberg EM Kjelby E Jørgensen HA. Sexual dysfunction and hyperprolactinemia in male psychotic inpatients: a cross-sectional study. Adv Urol. (2011) 2011:686924. 10.1155/2011/686924 22190916 PMC3235493

[44] Xiang YT Wang CY Si TM Lee EH He YL Ungvari GS. The low frequency of reported sexual dysfunction in Asian patients with schizophrenia (2001-2009): low occurrence or ignored side effect. Hum Psychopharmacol. (2011) 26:352–7. 10.1002/hup.1213 21751252

[45] Quinn-Nilas C Milhausen RR McKay A Holzapfel S. Prevalence and predictors of sexual problems among midlife Canadian adults: results from a national survey. J Sex Med. (2018) 15:873–9. 10.1016/j.jsxm.2018.03.086 29753802

[46] Yasui-Furukori N Fujii A Sugawara N Tsuchimine S Saito M Hashimoto K. No association between hormonal abnormality and sexual dysfunction in Japanese schizophrenia patients treated with antipsychotics. Hum Psychopharmacol. (2012) 27:82–9. 10.1002/hup.1275 22249970

[47] Trinchieri M Trinchieri M Perletti G Magri V Stamatiou K Cai T. Erectile and ejaculatory dysfunction associated with use of psychotropic drugs: a systematic review. J Sex Med. (2021) 18:1354–63. 10.1016/j.jsxm.2021.05.016 34247952

[48] Macdonald S Halliday J MacEWAN T Sharkey V Farrington S Wall S. Nithsdale schizophrenia surveys 24: sexual dysfunction. Case-control study Br J Psychiatry. (2003) 182:50–6. 10.1192/bjp.182.1.50 12509318

[49] Malik P Kemmler G Hummer M Riecher-Roessler A Kahn RS Fleischhacker WW. Sexual dysfunction in first-episode schizophrenia patients: results from European First Episode Schizophrenia Trial. J Clin Psychopharmacol. (2011) 31:274–80. 10.1097/JCP.0b013e3182199bcc 21508850

[50] Strauss GP Waltz JA Gold JM. A review of reward processing and motivational impairment in schizophrenia. Schizophr Bull. (2014) 40 Suppl 2:S107–16. 10.1093/schbul/sbt197 24375459 PMC3934394

[51] Takeda K Matsumoto M Ogata Y Maida K Murakami H Murayama K. Impaired prefrontal activity to regulate the intrinsic motivation-action link in schizophrenia. Neuroimage Clin. (2017) 16:32–42. 10.1016/j.nicl.2017.07.003 29071207 PMC5650579

[52] Yang Y Yang ZY Zou YM Shi HS Wang Y Xie DJ. Low-pleasure beliefs in patients with schizophrenia and individuals with social anhedonia. Schizophr Res. (2018) 201:137–44. 10.1016/j.schres.2018.05.018 29804927

[53] Najas-Garcia A Gómez-Benito J Huedo-Medina TB. The relationship of motivation and neurocognition with functionality in schizophrenia: a meta-analytic review. Community Ment Health J. (2018) 54:1019–49. 10.1007/s10597-018-0266-4 29605875

[54] Costa AM Lima MS Mari Jde J. A systematic review on clinical management of antipsychotic-induced sexual dysfunction in schizophrenia. São Paulo Med J. (2006) 124:291–7. 10.1590/S1516-31802006000500012 17262163 PMC11068300

